# Nanostring-based screening for tyrosine kinase fusions in inflammatory myofibroblastic tumors

**DOI:** 10.1038/s41598-020-75596-3

**Published:** 2020-10-30

**Authors:** Taisei Kurihara, Yoshiyuki Suehara, Keisuke Akaike, Takuo Hayashi, Shinji Kohsaka, Toshihide Ueno, Nobuhiko Hasegawa, Tatsuya Takagi, Keita Sasa, Taketo Okubo, Youngji Kim, Hiroyuki Mano, Takashi Yao, Kazuo Kaneko, Tsuyoshi Saito

**Affiliations:** 1 Department of Human Pathology, Graduate School of Medicine, Juntendo University, 2-1-1 Hongo, Bunkyo-Ku, Tokyo, 113-8421 Japan; 2 Department of Orthopedic Surgery, Juntendo University School of Medicine, Tokyo, 113-8421 Japan; 3 Intractable Disease Research Center, Graduate School of Medicine, Juntendo University, Tokyo, 113-8421 Japan; 4 Division of Cellular Signaling, National Cancer Center Research Institute, Tokyo, 104-0045 Japan

**Keywords:** Cancer, Genetics, Diseases, Medical research, Molecular medicine, Oncology, Pathogenesis

## Abstract

Gene expression imbalances were measured for tyrosine kinase (*TK*) genes using Nanostring in 19 samples of inflammatory myofibroblastic tumor (IMT). All cases were immunohistochemically stained with anaplastic lymphoma kinase (ALK) and pan-tropomyosin-related-kinase (pan-Trk) antibodies. Five cases with imbalanced *ALK* expression, reported with Nanostring, were tested using fluorescence in situ hybridization (FISH); two cases with imbalanced neurotrophic tyrosine receptor kinase 3 (*NTRK3*) expression were tested using reverse transcription-polymerase chain reaction (RT-PCR). One case with imbalanced expression for ROS proto-oncogene 1 (*ROS1*) was tested using RNA sequencing and RT-PCR. TK fusions were detected in all cases with imbalanced *TK* expression. RNA sequencing detected a *FN1–ROS1* fusion gene in an adult IMT case. IMT with *ALK* rearrangement showed myofibroblast-dominant features. IMT with *ETV6–NTRK3* fusion showed prominent lymphoplasmacytic infiltration with scattered myofibroblasts. Pan-Trk IHC revealed only scattered positively stained cells in IMT with *ETV6–NTRK3* fusion gene. *ROS1*-positive IMT showed myofibroblast-dominant features.

## Introduction

Inflammatory myofibroblastic tumor (IMT) is a rare mesenchymal tumor that can occur at any age; however, it is most common among the children and young adults^1^. Although IMT can occur at any anatomical site, the lungs, abdomen/pelvis, and retroperitoneum are frequently affected. The tumor is histologically characterized by spindle myofibroblastic cell proliferation with a lymphoplasmacytic inflammatory infiltrate^2^. The standard IMT treatment is surgical resection; however, treatment options are limited for patients with unresectable tumors or at advanced stage of the disease.

A clonal rearrangement of chromosome 2p23 involving the anaplastic lymphoma kinase (*ALK*) gene is detected in approximately 50% of the IMT cases, resulting in *ALK* overexpression and hyperactivation^3^. Furthermore, recent studies have reported novel fusion genes, such as ROS proto-oncogene 1 (*ROS1*)^4^, platelet-derived growth factor receptor beta (*PDGFRB*), and neurotrophic tyrosine receptor kinase 3 (*NTRK3*) in these tumors^2,5,6^. Identification of these fusion genes involving tyrosine kinases (*TK*) in IMT opened a new therapeutic option for IMT patients, especially for those with advanced-stage tumors.

However, given the histological diversity of IMT, even experienced pathologists face difficulty in reaching a definite diagnosis. Thus, identification of the histological cues using appropriate molecular pathology techniques holds significant importance in the correct diagnosis of IMT. Here, we comprehensively screened the imbalances of *TK* gene expression to identify the novel fusions in IMTs.

## Materials and methods

### Case selection

We collected information regarding cases diagnosed as either IMT or inflammatory pseudotumor (IPT) from the files at the Pathology Department at Juntendo University Hospital, from 2008 to 2018. IMTs were diagnosed based on the histological and immunohistochemical characteristics described in the WHO Classification of Tumours^1^. Immunoglobulin G4 (IgG4)-related sclerosing disease was excluded using immunoglobulin G (IgG) and IgG4 immunohistochemical staining, together with available serum IgG4 data. Infectious lesions, including granulomatous lesions, were also excluded. As a result, a total of 19 cases, including one pediatric case, were diagnosed with IMT. These cases were numbered as IMT#1–19. All patients provided informed consent before the surgeries in accordance with the institutional review board policy.

### Immunohistochemistry (IHC)

IHC was performed for all cases using the antibodies described in Table 1. A pediatric soft tissue tumor with an *LMNA-NTRK1* fusion^7^ and a case of salivary gland secretory carcinoma with *ETV6-NTRK3* (formerly MASC)^8^ fusion were used as positive controls for pan-Trk IHC.

**Table 1:** Immunohistochemical antibodies

Antibody	Host	Clonality	Activation	Buffer	Dilution	Source
ALK(D5F3)	Rabbit	Monoclonal	98 °C, 45 min	Tris EDTA	1:250	Cell Signaling Technlogy
Pan Trk(EPR17341)	Rabbit	Monoclonal	100 °C, 30 min	Tris EDTA	1:250	Abcam
Desmin	Mouse	Monoclonal	Roche	CC1	1:100	Leica
SMA	Mouse	Monoclonal	-	-	1:200	DAKO
M-actin	Mouse	Monoclonal	-	-	1:100	DAKO
h-caldesmon	Mouse	Monoclonal	98 °C, 30 min	Tris EDTA	1:1	DAKO
IgG	Mouse	Monoclonal		Citrate (pH6.0)	1:1000	Leica
IgG4	Mouse	Monoclonal	Proteinase K		1:4000	Southern Biotech

### RNA extraction

RNA was extracted from formalin-fixed paraffin-embedded (FFPE) samples using the RNeasy FFPE Kit (QIAGEN, Hilden, Germany). The primary tumor sample was used in cases of recurrence.

### Nanostring analysis

The Nanostring (NanoString Technologies, Inc., Seattle, WA, USA) procedure was performed (probe set described in Supplementary Table 1) to target 90 different *TK* and three serine/threonine kinases (*BRAF*, *ARAF*, and *CRAF*). At least two probes, spanning the exons and 100 bp in size were designed for each gene. One was designed against the 5′-terminal sequence and another against the 3′-terminal sequence. Briefly, 400 ng of ribonucleic acid (RNA) was hybridized to the probes (a reporter probe and a capture probe) at 65 °C for 18–24 h using a thermal cycler. Samples were inserted into the nCounter Prep Station to remove excess probes, purify, and immobilize the sample on the internal surface of a sample cartridge for 3 h. Finally, the sample cartridge was transferred to the nCounter Digital Analyzer, where color codes were counted and tabulated for each target molecule. The expression number for the base sequence of the probe part was analyzed using nSolver Analysis Software Version 4.0 (https://www.nanostring.com/products/analysis-software/nsolver). Raw data were statistically analyzed and plotted on graphs [X-axis: log_2_(5′-end expression), Y axis: log_2_(3′-end expression)] with 95% confidence interval (CI) lines. The case was deemed to have imbalanced gene expression if one end expression was more than tenfold higher than the other end expression or out of the 95% CI area of the graph.

### RNA sequencing

Total RNA was extracted from FFPE samples using the RNeasy FFPE Kit (Qiagen, Hilden, Germany) before treatment with deoxyribonuclease 1 (DNase 1) (Thermo Fisher Scientific; Waltham, MA, USA). The RNA-Seq library was prepared using the NEBNext Ultra Directional RNA Library Prep Kit (New England Bio Labs Inc., Tokyo, Japan) and according to the manufacturer’s protocol. Next-generation sequencing (NGS) was carried out from both ends of each cluster using the HiSeq2500 platform (Illumina, San Diego, CA, USA).

### Reverse transcription-polymerase chain reaction (RT-PCR)

We performed RT-PCR to confirm the fusion genes *ETV6–NTRK3* or *FN1–ROS1* using the PCR SuperMix (Thermo Fisher Scientific, Commonwealth, MA, USA). RNA quality was determined using a Nanodrop, and total RNA was reverse-transcribed to complementary DNA (cDNA) using the SuperScriptIV First-Strand Synthesis Kit (Invitrogen). RT-PCR was performed using the Platinum Green Hot Start PCR Master Mix (Invitrogen) for 40 cycles at 55 °C annealing temperature with the following primer pairs. The *ETV6–NTRK3* primer sequences were 5′-ACCACATCATGGTCTCTGTCTCCC-3′ and 5′-CATCGTGCTGAAGCGAGAACTG-3′^8^. *FN1–ROS1* primer sequences were 5′-CCATAAAGGGCAACCAAGAG-3′ and 5′-CAGTGGGAGAAAGCTGAAGAT-3′. Glyceraldehyde 3-phosphate dehydrogenase (GAPDH) expression was used as an RNA quality control using the following primers: 5-GAAGGTGAAGGTCGGAGTC-3 and 5- GAAGATGGTGATGGGATTT-3′.

### Fluorescence in situ hybridization (FISH)

In cases of imbalanced *ALK* expression, FISH was performed on 4-μm-thick unstained tissue sections (Vysis LSI ALK Dual-Color, BreakApart Rearrangement Probe Kit.). The FISH signals were scored by evaluating 50 tumor cell nuclei per case. A split signal was defined by 5′ and 3′ signals at a distance greater than a single signal width, and signals separated by a distance less than a single signal width were regarded as fused signals. Tumor cells showing split signals or isolated 3′ signals were concluded to have *ALK* rearrangements. We interpreted the result as FISH-positive, if > 15% tumor cells showed gene rearrangement^6^.

### Ethical standards

This study was reviewed and approved by the Juntendo University School of Medicine Institutional Review Board (#2019-034).

## Results

### Nanostring assays for *TK* fusion screening

**Figure 1:**
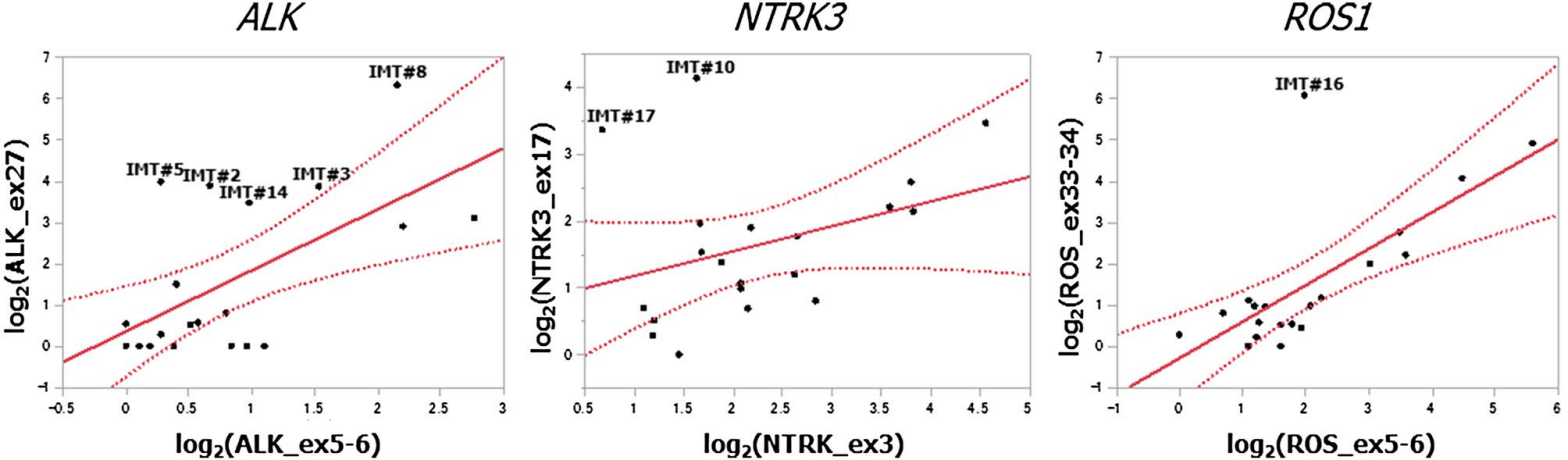
Graphs for *ALK*, *NTRK3*, and *ROS1* expression after normalization using nSolver Analysis software Version 4.0 in all cases. X-axis: gene expression measured by a probe located on the 5′-end, Y-axis: gene expression measured by a probe located on the 3′-end. The red reference line represents twofold standard deviation (SD). The expression data was analyzed using the nSolver Analysis Software Version 4.0 (https://www.nanostring.com/products/analysis-software/nsolver).

The clinicopathological features, including IHC findings of 19 IMT cases, are summarized in Table 2. We created graphs with a scatter plot for each tyrosine kinase receptor (TKR) in these cases. The Nanostring result quantifies the number of expressions (Supplementary Table 2). Imbalanced expression was defined if 3′-end expression was more than tenfold higher than 5′-end expression or lying out of the 95% CI area of the graph. This definition revealed imbalanced *TK* gene expression in *ALK, ROS1,* and *NTRK3*, which had been previously reported, and we conducted subsequent analysis on these three genes. Finally, five *ALK*, one *ROS1*, and two *NTRK3* cases were identified as imbalanced cases (Fig. 1).

**Table 2:** Clinicopathological and molecular findings in IMTs.

No	Age	Sex	Location	Size(mm)	Outcomes	Imbalance of TK	Fusion/ rearrangement	Evaluation Methods	Original diagnosis	Dominant histology	IHC							IgG(mg/dL) N.R 870-1700	IgG4(mg/dL)N.R. 4.5-117
											Desmin	SMA	M-actin	h-caldesmon	IgG4/HPF	IgG/HPF	IgG4/IgG		
vIMT#1	59	M	Intraorbital	27 × 15	NED(130mos)	(-)	(-)	IHC(-)	IMT	spindle cells, bizarre cells	−	+	+	focal+	1	200	0.5	856	126
IMT#2	44	M	Right lung	20 × 20	NED(57mos)	***ALK***	(+)	ALK FISH(+), ALK IHC(+)	IMT	spindle cells, ossification	−	+	+	focal+	0	0	0.0	N/A	N/A
IMT#3	60	M	Right lung	22 × 20	NED(6mos)	***ALK***	(+)	ALK FISH(+), ALK IHC(+)	IMT	spindle cells, foamy cells	focal+	focal+	focal+	weak+	5	200	2.5	821	N/A
IMT#4	44	F	Left lung	16 × 16	NED(118mos)	(-)	(-)	IHC(-)	IMT	lymphoplasmacytic infiltration	focal+	+	+	focal+	20	200	10.0	1243	966
IMT#5	42	M	Left lung	9 × 9	NED(39mos)	***ALK***	(+)	ALK FISH(+), ALK IHC(+)	IMT	spindle cells, foamy cells	focal+	+	+	focal+	10	40	25.0	N/A	N/A
IMT#6	34	M	Mediastinum	80 × 63	AWD (12mos)	(-)	(-)	IHC(-)	IPT	lymphoplasmacytic infiltration	focal+	focal+	focal+	−	5	150	3.3	N/A	N/A
IMT#7	58	M	Inguinal canal	15 × ×6	Dead*(7mos)	(-)	(-)	IHC(-)	IMT	spindle cells, prominent nucleoli	focal+	+	+	+	35	130	26.9	N/A	N/A
IMT#8	22	F	Bronchus	14 × 11	NED(7mos)	***ALK***	(+)	ALK FISH(+), ALK IHC(+)	IMT	spindle cells, foamy cells	−	+	focal+	−	3	100	3.0	N/A	N/A
IMT#9	75	F	Left ethmoid sinus	27 × 20	AWD(109mos)	(-)	(-)	IHC(-)	IPT	lymphoplasmacytic infiltration	focal+	+	+	focal+	5	200	2.5	1314	N/A
IMT#10	41	F	Right lung	32 × 30	NED(46mos)	***NTRK3***	***ETV6-NTRK3(+)ETV6-NTRK3(+)***	pan-trk IHC(+), RT-PCR(+)	IPT	lymphoplasmacytic infiltration	−	+	focal+	focal+	3	200	1.5	N/A	N/A
IMT#11	46	M	Liver	20 × 10	NED(2mos)	(-)	(-)	IHC(-)	IPT	lymphoplasmacytic infiltration	+	+	+	focal+	60	180	33.3	N/A	N/A
IMT#12	60	M	Liver	14 × 14	NED(69mos)	(-)	(-)	IHC(-)	IPT	lymphoplasmacytic infiltration	focal+	+	+	+	20	180	11.1	N/A	N/A
IMT#13	30	F	Liver	9 × 10	NED(107mos)	(-)	(-)	IHC(-)	IPT	lymphoplasmacytic infiltration	−	+	+	focal+	10	200	5.0	N/A	N/A
IMT#14	4	M	Ileum	60 × 50	NED(74mos)	***ALK***	(+)	ALK FISH(+), ALK IHC(+)	IPT	myxoid, hypocellular, prominent nucleoli	+	+	focal+	focal+	3	100	3.0	N/A	N/A
IMT#15	60	F	Orbit	20 × 14	NED(9mos)	(-)	(-)	IHC(-)	IPT	lymphoplasmacytic infiltration	−	+	focal+	focal+	5	150	3.3	N/A	N/A
IMT#16	55	M	Left paratid gland	21 × 15	NED(8mos)	***ROS1***	***FN1-ROS1(+)***	RNA sequence	IPT	spindle cells, prominent nucleoli	−	+	+	focal+	15	80	18.8	N/A	N/A
IMT#17	56	M	Left lung	27 × 23	NED (29mos)	***NTRK3***	***ETV6-NTRK3(+)***	pan-trk IHC(+), RT-PCR(+)	IPT	lymphoplasmacytic infiltration	−	+	+	focal+	15	200	7.5	N/A	N/A
IMT#18	64	M	Orbit	35 × 33	AWD (129mos)	(-)	(-)	IHC(-)	IPT	lymphoplasmacytic infiltration	−	+	+	focal+	1	50	2.0	N/A	N/A
IMT#19	38	F	Anterior mediastinum	120 × 26	AWD (33mos)	(-)	(-)	IHC(-)	IPT	lymphoplasmacytic infiltration	−	+	focal+	focal+	5	100	5.0	N/A	N/A

*Died 10 days after surgery due to sepsis.

*N.R.* Normal range.

Numbers of IHC of IgG and IgG4 were counted on the high-power field (HPF).

### Confirmatory studies

Further analyses (FISH, RT-PCR, IHC, and RNA sequencing) were performed for cases showing imbalanced gene expression based on Nanostring results.

### FISH

**Figure 2:**
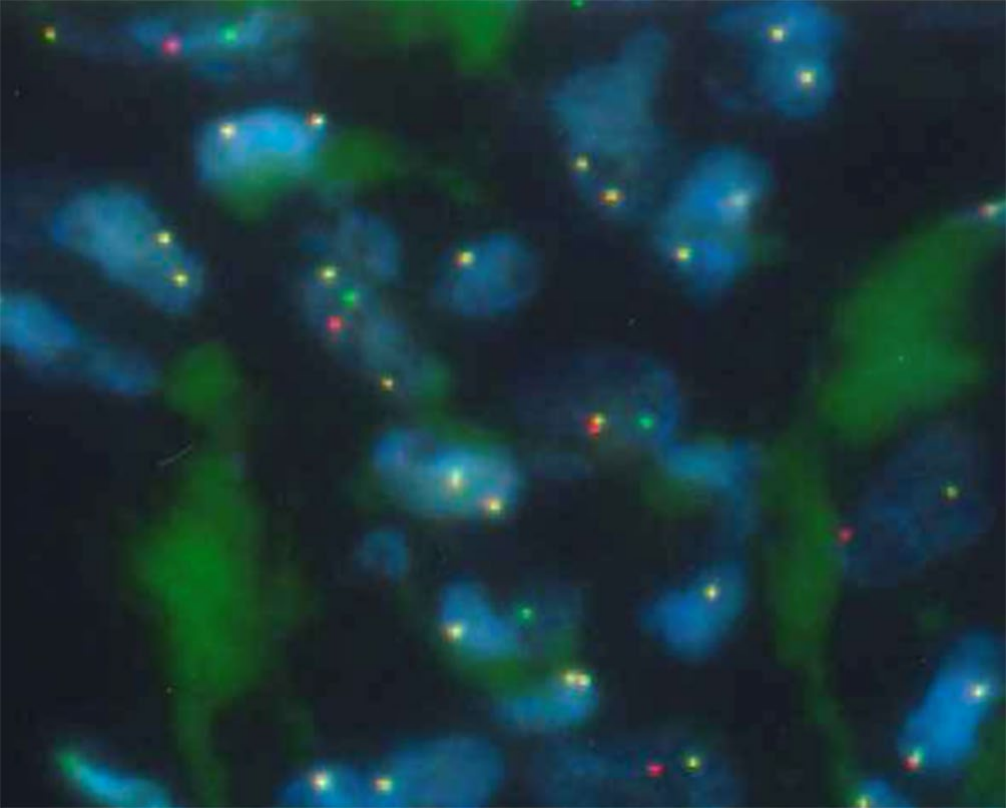
FISH for *ALK* rearrangement. The majority of tumor cells show a split signal pattern of one green signal (*ALK* 5′-end) and one red signal (*ALK* 3′-end).

We found *ALK* rearrangement in all five *ALK* imbalanced cases. One case occurred in the ileum, and the remaining four cases occurred in the lungs. *ALK* rearrangements were detected in all five cases (Fig. 2).

### IHC

**Figure 3:**
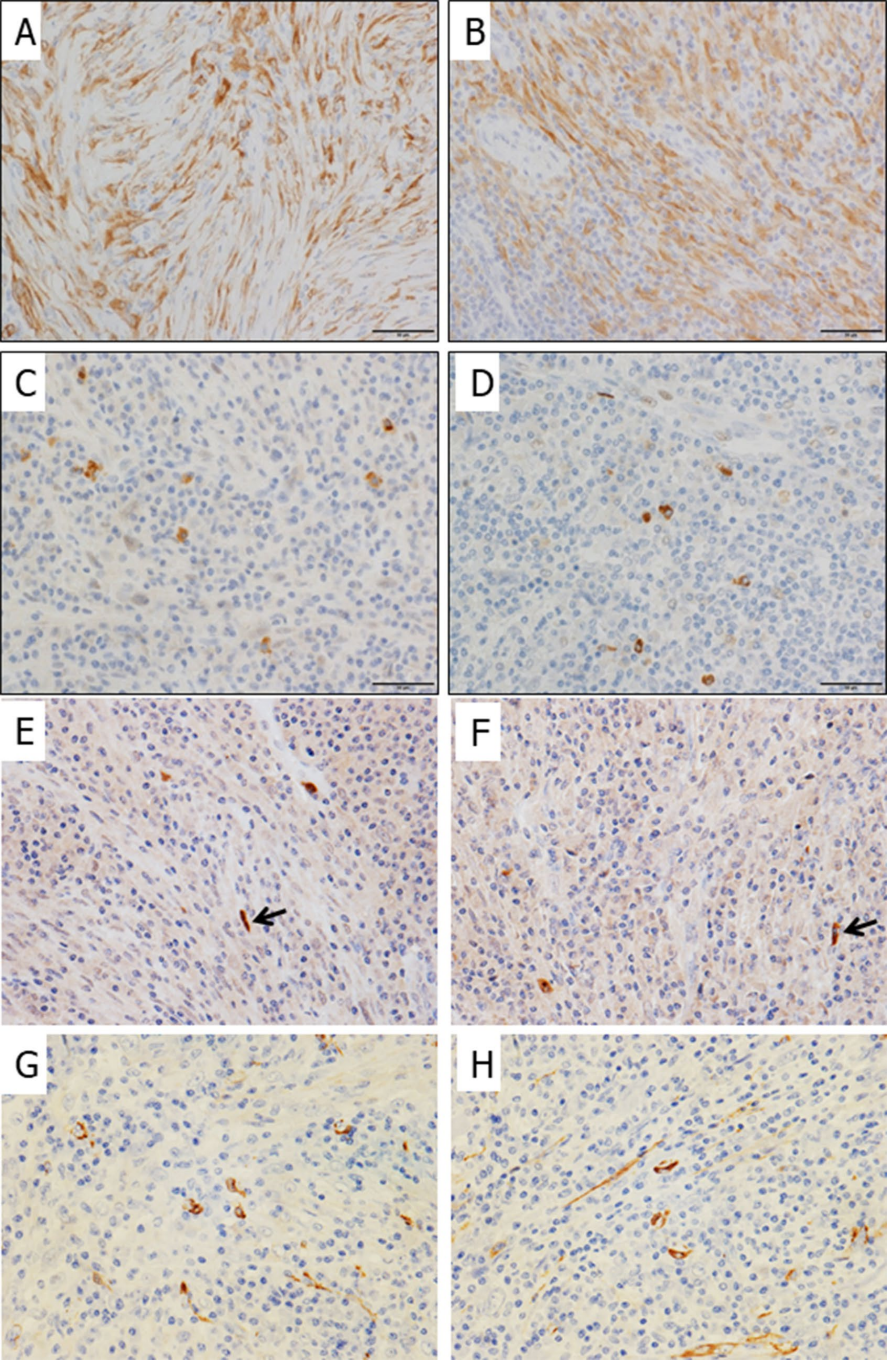
Immunohistochemistry for ALK and pan-Trk. Strong *ALK* expression was observed. (**A**: IMT#2,** B**: IMT#3). Only scattered positively stained cells within lymphoplasmacytic backgrounds were observed in two IMTs with *NTRK3* fusion (**C**: IMT#10, **D**: IMT#17). Spindle-shaped cells also showed positive staining for pan-Trk IHC (**E**: IMT#10, **F**: IMT#17). SMA IHC staining showed scattered positively stained cells among morphologically indistinguishable lymphoplasmacytic cells in addition to the spindle-shaped cells (**G**: IMT#10, **H**: IMT#17). (**A**–**H**): 400 ×  magnification.

ALK IHC staining was performed in all cases and revealed five positive cases (Fig. 3A,B). All five cases showed imbalanced *ALK* expression based on Nanostring data and were FISH-positive. Pan-Trk staining was also performed in all cases, including the two cases in which the *NTRK3* fusion gene was confirmed using RT-PCR. Only scattered positively stained cells within lymphoplasmacytic backgrounds were observed in two IMTs with *NTRK3* fusion (Fig. 3C–F). Smooth muscle actin (SMA) IHC staining also highlighted the scattered positively stained spindle-shaped cells (Fig. 3G,H). A pediatric soft tissue tumor with *LMNA–NTRK1* showed diffuse and strong cytoplasmic staining for pan-Trk (Supplementary Fig. 1A). In contrast, a salivary gland secretory carcinoma with *ETV6–NTRK3* fusion primarily exhibited typical nuclear staining with weak cytoplasmic staining for pan-Trk (Supplementary Fig. 1B).

### RNA sequencing

RNA was sequenced in the single case with *ROS1* imbalance. As a result, a *FN1-ROS1* fusion gene was discovered.

### RT-PCR

**Figure 4:**
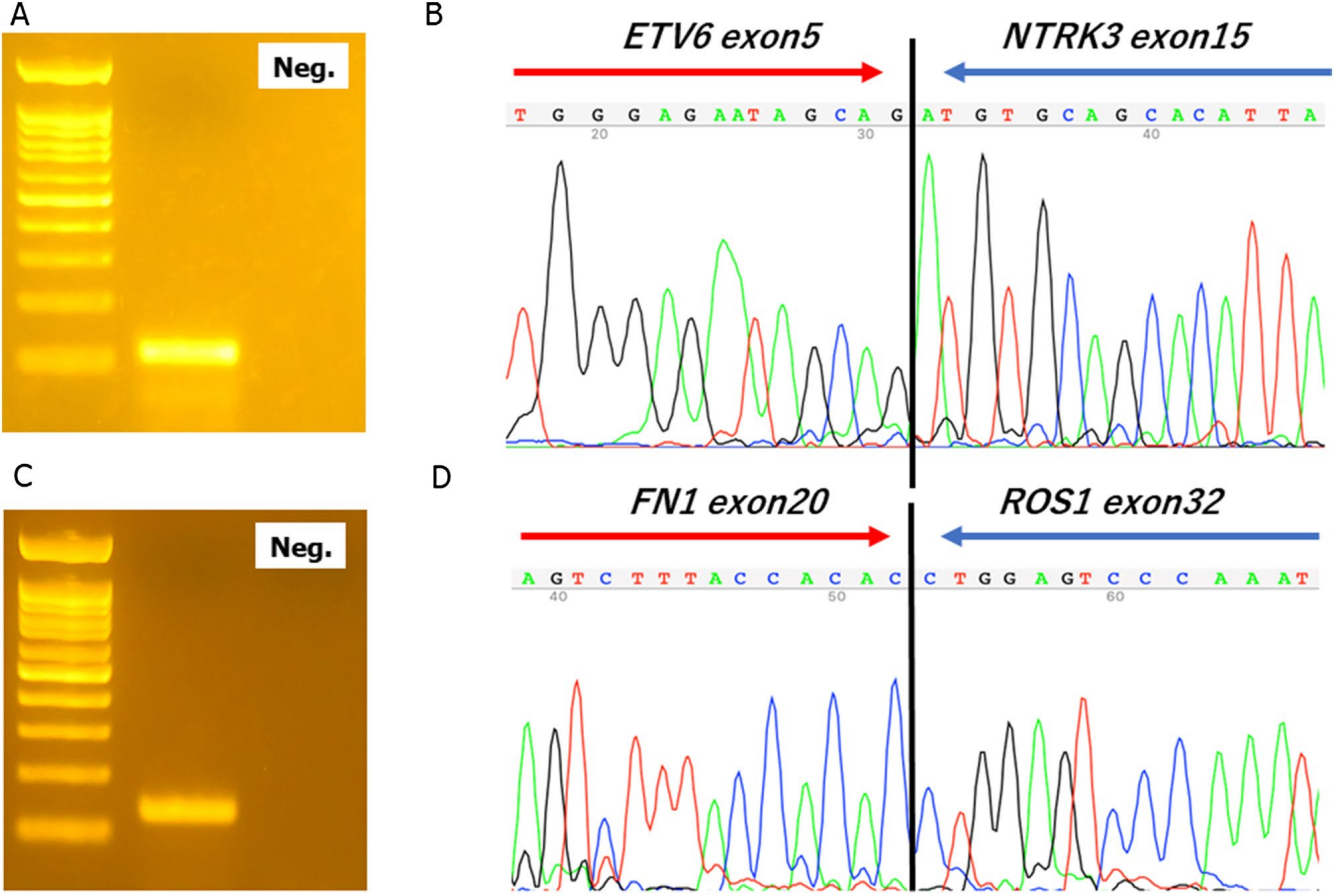
RT-PCR and Sanger sequencing. (**A**) A 110 bp RT-PCR product was detected using the *ETV6–NTRK3* fusion primer pair. (**B**) Sanger sequencing demonstrates that exon 5 of *ETV6* is fused to exon 15 of *NTRK3* in IMT#10. (**C**) A 131 bp PCR product was detected using the *FN1–ROS1* fusion primer pair. (**D**) Sanger sequencing demonstrates that exon 20 of *FN1* is fused to exon 32 of *ROS1* in IMT#16. Neg.: Negative control without template cDNA.

*ETV6–NTRK3* fusion was detected using RT-PCR in two IMT cases with imbalanced *NTRK3* expression. Both cases had the same fusion gene with *ETV6* exon 5 fused to *NTRK3* exon 15 based on Sanger sequencing (Fig. 4A,B). The first case was reported in a 41-year-old woman with IMT occurring in the right lung (IMT#10). Another example is of a 56-year-old man with IMT occurring in the left lung (IMT#17). *FN1–ROS1* fusion was confirmed by RT-PCR and direct sequencing in the *ROS1* imbalance case (Fig. 4C,D).

### Histology of IMT with TK fusion

**Figure 5:**
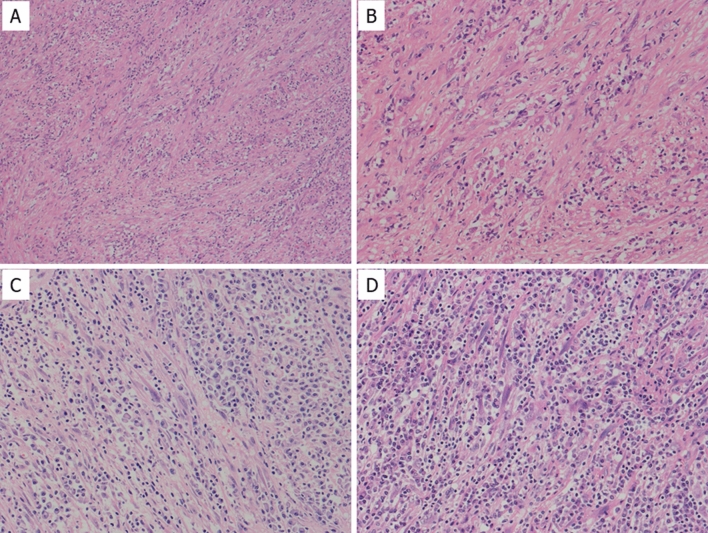
Histological features of IMT with TK fusion/rearrangement. *ROS-1*-positive IMT showing admixture of spindle-shaped cells with prominent nucleoli and lymphoplasmacytic inflammatory cells within collagenous background (**A**,**B**). Two cases with *NTRK3* fusion showing prominent lymphoplasmacytic infiltration with scattered spindle-shaped myofibroblasts (**C**: IMT#10, **D**: IMT#17). (**A**): 100 × , (**B**–**D**): 200 × magnification.

The dominant histological features are described in Table 2. Among the seven cases originally diagnosed as IMT, tumors in six cases were mainly composed of proliferating spindle cells with inflammatory infiltrates. Only one of the seven cases showed prominent lymphoplasmacytic infiltration with myofibroblastic cells. Four out of five IMT with *ALK* rearrangements were originally diagnosed as IMT. In contrast, among the 12 cases originally diagnosed as IPT, only two cases showed spindle cell predominant features, but both cases had *TK* fusion/rearrangement. One case with *ALK* rearrangement showed hypocellular proliferation of the spindle-shaped cells with prominent nucleoli in the myxoid background. In another case, *ROS-1* positive IMT showed a histological admixture of spindle-shaped cells with prominent nucleoli and lymphoplasmacytic inflammatory cells within the collagenous background (Fig. 5A,B). Two cases with *NTRK3* fusion showed prominent lymphoplasmacytic infiltration with scattered spindle-shaped myofibroblasts (Fig. 5C,D).

## Discussion

IMT is currently classified as an intermediate and rarely metastasizing neoplasm composed of myofibroblasts accompanied by an inflammatory infiltrate containing plasma cells, lymphocytes, and eosinophils in varying proportions. Most patients with IMT are children, adolescents, or young adults, but the tumor can occur throughout life. IMT was regarded as an inflammatory tumor-like lesion under the name IPT, which showed histological diversity. The concept of IMT was proposed because of its invasive and metastatic nature. Since the introduction of IMT, various gene fusion studies have been conducted. Approximately 50%–70% of IMT have been reported to harbor *ALK* gene rearrangement, leading to a chimeric fusion protein formation detectable by IHC or FISH^2,9^. However, this frequency varies from study to study, probably due to histological diversity. In IMT, more than 10 different genes have been identified as *ALK* fusion partners that provide a strong promoter and an oligomerization domain, resulting in oncogenic activation of the *ALK* kinase^2^. Furthermore, recent studies in IMT have described novel fusions involving *ROS1*, *PDGFRB*, and *NTRK3* genes^2,4–6^. A recent study has demonstrated that all thoracic IMTs harbor *TK* abnormalities including *ALK, ROS1, NTRK3*, and rearranged during transfection (*RET)* TK fusions, and only one case had alternative *ALK* transcription initiation^10^.

We investigated the frequency of *TK* fusions in order to report a novel fusion in IMT. The Nanostring screening system employed in the study was based on the theory that a 5′-end/3′-end imbalance should be observed if fusion genes involving *TKR* were formed and maintained^11^. We found eight imbalanced cases using Nanostring and detected gene fusions/rearrangements in all of them. The imbalance criterion is a key factor in this assay. A previous study used 5′/3′ ratio below -4 on a log_2_ scale, which is consistent with the 5′/3′ ratio > 16-fold^11^. Another study used a 3′/5′ ratio > fivefold for ALK probes^10^. One of our two criteria was a 3′/5′ ratio > tenfold for each probe. We also employed another standard that used our accumulated Nanostring data set composed of more than 1000 sample data obtained using the same probe set. For this criterion, samples plotted out of the 95% CI area were considered significant, and all of these samples showed a 3′/5′ ratio > tenfold for the corresponding probe. Furthermore, the remaining cases lying within the 95% CI area showed 3′/5′ ratio < threefold. All the eight cases with *TK* fusions tested positive for both the criteria. Demonstrating the sensitivity of the scoring based on these two criteria, we detected only one case lying out of the 95% CI but negative for ALK IHC and FISH among all the collected samples. This case was not found positive upon screening with the tenfold increase criterion. Therefore, we believe that screening for a tenfold increase in case of 3′/5′ ratio would be more reliable. Regarding the correlation between IHC and Nanostring analysis, IHC was performed for all cases using ALK and pan-Trk antibodies, and all cases positive for either ALK or pan-Trk IHC showed imbalanced expression for *ALK* or *NTRK3* by Nanostring, respectively. It is well known that the IHC staining pattern and intensity highly depends on the staining conditions, such as antibody dilution, antigen retrieval method, and the incubation time. Here, IHC titration could be considered ideal because none of the cases negative for imbalance showed positive results for pan-Trk and ALK IHC.

There are many variations for fusion genes in terms of partners and junctions, so it is difficult to cover all the possible fusion genes using RT-PCR. We have set all the 3′-end probes based on the sequences after the tyrosine kinase domain (TKD) for each TKR, allowing us to efficiently detect the functioning imbalance. These probes were also designed to target the exon–exon boundaries, to reduce the risk of interfering signals from genomic deoxyribonucleic acid (DNA) and correctly reflect the gene expression status. Our series showed that only 42% of the IMT cases harbored gene fusion/rearrangement involving either *ALK, NTRK3*, and *ROS1*, but it has been shown that approximately 50%–70% of the IMTs harbor ALK fusion/rearrangement^2,9^. A racial difference might have influenced the frequency of fusion genes involving TKRs in the study IMT samples. A recent study has demonstrated that 82.5% of the Japanese IMT cases had rearrangements involving either *ALK, NTRK3*, or *ROS1*^12^, indicating that racial differences might be less likely to cause the low frequency of gene fusion/rearrangement. However, it has also been reported that pediatric cases frequently harbor *TK* fusions^3,13,14^. Our samples contained only one pediatric case, but one-third of the cases in a previous Japanese study were pediatric cases^12^. Therefore, the age difference might have affected the frequency of *TK* fusion/gene rearrangements. Furthermore, there also remains the possibility that IgG4-related sclerosing disease has not been completely excluded from the samples, since laboratory data on serum IgG4 levels were not available for most of the cases, although IgG and IgG4 IHC were performed for all cases. Interestingly, a recent study demonstrated that all thoracic IMTs harbor *TK* abnormalities, most of which are *TK* fusions^10^. In this study, *TK* fusions were detected in six of nine thoracic and two out of ten non-thoracic IMTs, respectively. We observed the same trend as that observed in the previous study^10^, and this difference was nearly statistically significant (*p* = 0.07).

Among the 19 IMT cases originally diagnosed as either IMT or IPT in this study, 7 cases were of IMT. FISH detected *ALK* gene rearrangements in four of these IMT cases (57.1%), which is consistent with the reported frequency of *ALK* fusion/rearrangements. The remaining 12 cases that have been originally diagnosed as IPT but revised as IMT by retrospective reviews, showed relatively few myofibroblasts with prominent inflammatory cells, including lymphocytes and plasma cells with hyalinized stroma. Only 4 of the 12 IPT cases harbored *TK* fusions (33.3%); however, interestingly, only one out of these four cases with *TK* fusions had *ALK* fusion/rearrangements. Thus, the relatively low frequency of *TK* fusions in our IMT samples may reflect the histological diversity in the lesions, and genetically unrelated histological mimics, especially in adults, that might have been included in the study samples, diluting the *TK* fusion frequency. Although a novel *FN1–ROS1* fusion gene was recently discovered in an infantile IMT case^4^, we found, for the first time, *FN1–ROS1* fusion in an adult IMT case, indicating that this fusion gene exists in both infant and adult patients. Although the practical scope of employing the Nanostring-based screening system in most hospitals is limited, the information is helpful for detecting *ROS1* fusion in IMT with routine molecular tests using RT-PCR. This could lead to a potential molecular target therapy. In the near future, IMT might be defined and reclassified based on molecular pathological information concerning *TK* fusion/alteration^10^. The imbalanced *TK* expression detection by Nanostring or even quantitative PCR (qPCR) can be useful for not just identifying true IMT but also in the future molecular classification of IMT, keeping aside the concerns related to the histological diversity within IMT.

IMT with *ETV6–NTRK3* fusion might have characteristic histological features. SMA staining confirmed that both the cases with *ETV6–NTRK3* fusion were histologically composed of prominent lymphoplasmacytic infiltration with scattered myofibroblasts. The observation is closely related to the finding that only focal staining was observed using pan-Trk IHC in the two IMT cases with *ETV6–NTRK3* fusion. In this regard, Alassiri et al. reported that IMT with *ETV6-NTRK3* fusion can show a wide variety of histological features such as loose fascicles of spindle cells with prominent plasmacytic infiltrate, myxoid background, and ganglion-like cells^5^. It has been reported that pan-Trk IHC is highly sensitive and specific for *NTRK3* fusion-positive tumor detection^15,16^. However, in comparison to *NTRK1-* and *NTRK2*-positive tumors, *NTRK3* fusion-positive tumors are less sensitive to pan-Trk IHC^17^. Here, pan-Trk IHC was also performed on *ETV6–NTRK3*-positive secretory carcinoma (formerly MASC) under the same IHC staining conditions, but mainly nuclear rather than cytoplasmic staining was observed. However, an *NTRK1* fusion tumor showed strong and diffuse cytoplasmic staining under the similar conditions (Supplementary Fig. 1). Although a recent study has demonstrated that nuclear and cytoplasmic staining patterns can be observed by pan-Trk IHC in *NTRK3* fusion-positive IMT^12^, it is important to know that *NTRK3* fusion-positive IMTs could also show a scattered nuclear/cytoplasmic staining pattern. This pattern is different from the diffused nuclear and poor cytoplasmic staining pattern of *ETV6–NTRK3*-positive secretory carcinoma (formerly MASC). Therefore, we shall conduct further studies based on this IHC finding, including RT-PCR and RNA sequencing. In addition, IMT cells with scattered staining pattern for pan-Trk IHC showed lymphoplasmacytic features, raising the possibility of being considered as a true IMT with *NTRK3* fusion. Most of these positively stained cells for pan-Trk IHC had oval nuclei, and could not be morphologically distinguished from lymphoplasmacytic cells, and seemed not to display myofibroblastic characteristics. However, small amounts of spindle-shaped cells also exhibited positive staining for pan-Trk IHC though less frequent. In addition, SMA IHC staining also highlighted scattered positively stained cells with myofibroblastic nature among cells with oval nuclei.

Finally, our screening system also detected imbalanced gene expression for the following *TK* genes, other than *ALK, ROS1,* and *NTRK3*: *CSK* in IMT#1 and 9, *LMTK3* in IMT#4, *LTK* in IMT#11 and 17, *MUSTK* in IMT#13, and *EPHA4* in IMT#18. We searched for *TK* fusion genes involving the above listed *TK* genes in PubMed. Since the previous studies including those employing NGS failed to identify fusion genes involving these TKR genes, we excluded *CSK, EPHA4, LMTK3, LTK*, and *MUSTK* from the targets. However, it remains to be proven whether the 11 cases deemed negative for *TK* fusion in this study were really fusion-negative.

In summary, all *ALK* rearranged cases were positive for ALK IHC, and all cases other than *ALK* rearranged/*NTRK3* fusion-positive cases were negative for pan-Trk and ALK IHC. Thus, this Nanostring-based screening system was both 100% sensitive and specific. Out of the 19 IMT cases, we identified 8 cases (42%) with *TK* fusions. We found *FN1–ROS1* in adult patients with IMT. We must keep in mind that *NTRK3* fusion-positive IMTs frequently show scattered cytoplasmic staining pattern for pan-Trk IHC, which at times may be difficult to interpret. The Nanostring-based screening system is useful for detecting *TK* fusions, especially *NTRK3* fusions.

## Supplementary information

Supplementary Information 1.

Supplementary Information 2.

Supplementary Information 3.

Supplementary Information 4.
