# Supplementary figures and images for: Nanostring-based screening for tyrosine kinase fusions in inflammatory myofibroblastic tumors
Source: Sci Rep. 2020 Oct 30;10:18724. doi: 10.1038/s41598-020-75596-3 (PMC7603320; doi:10.1038/s41598-020-75596-3)

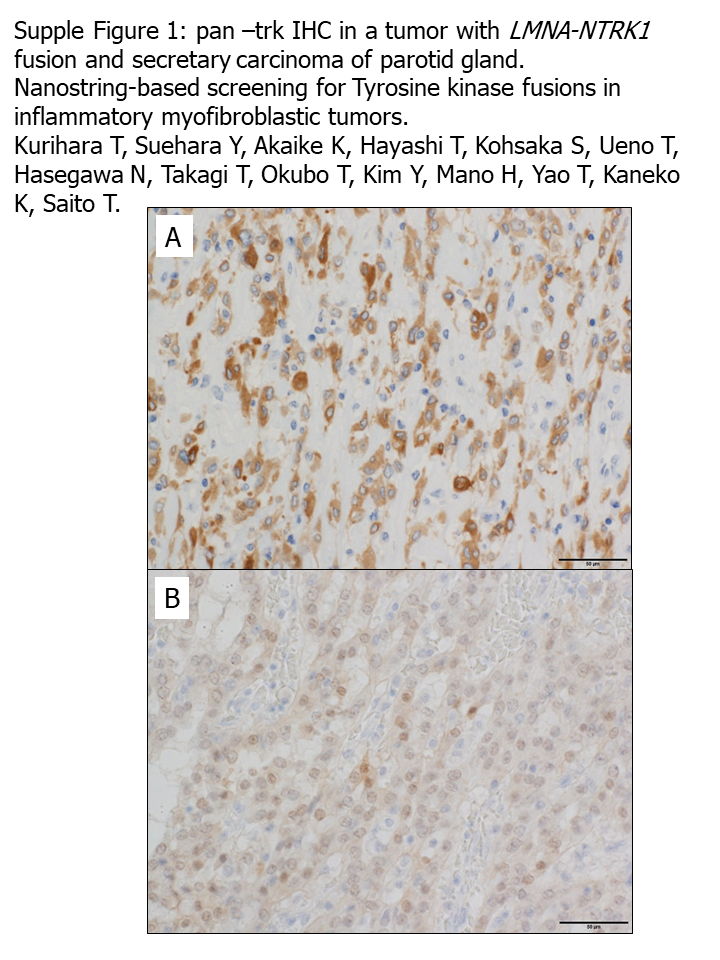

Supplement: Supplementary file 1 — Supplementary Information 1. [file 41598_2020_75596_MOESM1_ESM.tif]
