# Supplementary material for: Nanostring-based screening for tyrosine kinase fusions in inflammatory myofibroblastic tumors
Source: Sci Rep. 2020 Oct 30;10:18724. doi: 10.1038/s41598-020-75596-3 (PMC7603320; doi:10.1038/s41598-020-75596-3)
Supplement: Supplementary file 4 — Supplementary Information 4. [file 41598_2020_75596_MOESM4_ESM.docx]

**Supplementary Fig. 1**: (A) A pediatric soft tissue tumor with *LMNA–NTRK1* showing diffuse and strong cytoplasmic staining for pan-Trk. (B) Another positive control, a salivary gland secretory carcinoma with *ETV6–NTRK3* fusion, showing very weak cytoplasmic staining for pan-Trk.
